# Spin-dependent electrocatalysis

**DOI:** 10.1093/nsr/nwae314

**Published:** 2024-09-05

**Authors:** Zhengjie Chen, Xiaoning Li, Hao Ma, Yuwei Zhang, Jing Peng, Tianyi Ma, Zhenxiang Cheng, Jose Gracia, Yuanmiao Sun, Zhichuan J Xu

**Affiliations:** Faculty of Materials Science and Energy Engineering, Shenzhen University of Advanced Technology, Shenzhen 518107, China; School of Materials Science and Engineering, Nanyang Technological University, Singapore 639798, Singapore; School of Science, RMIT University, Melbourne 3000, Australia; Institute of Technology for Carbon Neutrality, Shenzhen Institute of Advanced Technology, Chinese Academy of Sciences, Shenzhen 518055, China; School of Materials Science and Engineering, Nanyang Technological University, Singapore 639798, Singapore; Faculty of Materials Science and Energy Engineering, Shenzhen University of Advanced Technology, Shenzhen 518107, China; Institute of Technology for Carbon Neutrality, Shenzhen Institute of Advanced Technology, Chinese Academy of Sciences, Shenzhen 518055, China; School of Science, RMIT University, Melbourne 3000, Australia; Institute for Superconducting and Electronic Materials (ISEM), Faculty of Engineering and Information Sciences, Innovation Campus, University of Wollongong, North Wollongong 2500, Australia; MagnetoCat SL, General Polavieja 9 3I, Alicante 03012, Spain; Faculty of Materials Science and Energy Engineering, Shenzhen University of Advanced Technology, Shenzhen 518107, China; Institute of Technology for Carbon Neutrality, Shenzhen Institute of Advanced Technology, Chinese Academy of Sciences, Shenzhen 518055, China; School of Materials Science and Engineering, Nanyang Technological University, Singapore 639798, Singapore; Center for Advanced Catalysis Science and Technology, Nanyang Technological University, Singapore 639798, Singapore

**Keywords:** spin-dependent electrocatalysis, spin configuration, spin-related features, characterization techniques, manipulation strategies

## Abstract

The shift towards sustainable energy requires efficient electrochemical conversion technologies, emphasizing the crucial need for robust electrocatalyst design. Recent findings reveal that the efficiency of some electrocatalytic reactions is spin-dependent, with spin configuration dictating performance. Consequently, understanding the spin's role and controlling it in electrocatalysts is important. This review succinctly outlines recent investigations into spin-dependent electrocatalysis, stressing its importance in energy conversion. It begins with an introduction to spin-related features, discusses characterization techniques for identifying spin configurations, and explores strategies for fine-tuning them. At the end, the article provides insights into future research directions, aiming to reveal more unknown fundamentals of spin-dependent electrocatalysis and encourage further exploration in spin-related research and applications.

## Teaser

This review presents the current understanding of spin-dependent electrocatalysis, highlighting key aspects such as spin features in electrocatalysts, techniques for spin characterization, strategies for spin manipulation, as well as the challenges and future perspectives in this emerging field.

## INTRODUCTION

The reliance on fossil fuels not only leads to a dwindling depletion of reserves and raised concerns of energy security, but also significantly contributes to global climate change through vast emissions of carbon dioxide and greenhouse gases [1]. This situation underscores the urgent need to shift towards renewable energy sources as a promising strategy for sustainable development. In this vein, electrocatalysis plays a crucial role in energy conversion by utilizing green electricity as the driving force, which can be sustainably generated via renewable sources such as wind, tidal, and geothermal energy [2–4]. The efficiency of electrocatalysis is primarily determined by two fundamental aspects: the thermodynamic interactions between catalyst and adsorbate, and the kinetics of electron transfer [5,6]. Hence, to realize superior electrocatalytic efficiency, both thermodynamic and kinetic aspects should be addressed. This entails fine-tuning the chemisorption energies of key reaction intermediates for thermodynamic improvements, and enhancing electron transfer from adsorbed reactants/intermediates to active sites and the charge transport within the catalyst for kinetic optimization [7,8].

Recent theoretical and experimental advances have disclosed the non-negligible contribution of spin-related features in influencing the thermodynamics and kinetics of electrochemical reactions [9–11]. Changes in these features can alter spin/charge density, modify electronic exchange interactions among atoms, and influence both electron spin and the material's magnetic properties [12–14], thereby affecting electrocatalytic activities. Particularly, for the reactions that do not conserve spin, such as oxygen evolution reaction (OER) and oxygen reduction reaction (ORR) [15,16], the spin-related features demonstrate straightforward influences in the efficiency of electrocatalysis. In these reactions, the O_2_ molecule, either as a product in OER or as a reactant in ORR, has two unpaired electrons on the π* orbitals with spin aligned in parallel, manifesting a triplet ground state. In contrast, the OH^–^ species involved in these reactions are in a singlet state [17]. According to the spin conservation rule, reactions involving reactants and products with different spin quantum numbers should be prohibitive in theory, or requiring extra energy for a spin flip. However, both the OER and ORR can proceed efficiently with the assistance of suitable electrocatalysts, particularly those with specific spin configuration that can facilitate this spin transition. Another spin-mediated promotion in electrocatalysis has been proposed by Nørskov and his team. Employing density functional theory (DFT) calculations, they found a novel spin-promoted effect in magnetic catalysts for ammonia synthesis, in which the enhanced catalytic activity on Co-based magnetic catalysts correlates directly with a reduction in the spin magnetic moments of the Co atoms. This anomalous spin-promoted effect significantly lowers the transition state energy barrier for N–N dissociation due to spin polarization, thereby enhancing catalytic efficiency [18]. The spin-enhanced mechanism has been further demonstrated by Chorkendorff *et al.* By introducing heterometallic atoms bound to the active sites on the catalyst surface, they observed that suppressing the magnetism of the catalyst may be a key to lower temperature operation for ammonia synthesis. Specifically, using lanthanum to quench the magnetic moment of adjacent cobalt centers enhances the cobalt's catalytic activity for nitrogen cleavage well below the temperature where reaction occurs. This innovative approach offers a promising avenue to take advantage of spin interactions for optimizing catalyst design [19]. Therefore, exploring the spin configuration and related physicochemical property of electrocatalysts is beneficial for a comprehensive understanding of spin-dependent electrocatalysis.

To fully comprehend spin-dependent electrocatalysis, it is essential to understand the fundamental principles that dominate spin-related features of electrocatalysts. Basically, the spin-related features primarily emerge from the occupancy of atomic orbitals and the orientation of electron spins [20]. Variations in orbital occupancy give rise to different spin states, thereby establishing distinct short-range atomic exchange interactions; while the orientation of electron spins can lead to diverse band structures, magnetic orderings, and electrical conductivities in electrocatalysts [21,22]. Thus, a thorough understanding of these spin-related features is critical as the first step in comprehending spin-dependent electrocatalysis. Furthermore, to uncover the mechanism through which spin-related features influence electrocatalytic reactions, an accurate examination and analysis of the electrocatalysts’ spin configuration is also necessary. This calls for the mastery of characterization technologies that can map out both localized and extensive spin configurations. Notably, recent advancements in theoretical approaches, both quantum computations and conceptual analysis, have become invaluable tools for analyzing spin-related reaction mechanisms [21,23]. These approaches serve as an effective complement by offering insights that bridge the gap between theoretical concepts and practical observations [24,25], and their synergy is pivotal in deepening our understanding of spin-dependent electrocatalysis. Finally, to further improve the efficiency of spin-dependent electrocatalysis, mastering the strategies that are effective in fine-tuning the spin configurations of electrocatalysts is crucial [26]. Basically, the degree to which a material's spin configuration can be adjusted dictates the extent to which spin-dependent phenomena can be leveraged for enhanced electrocatalytic activity. Thus, staying informed about the latest advancements in spin configuration manipulation techniques is essential for optimal electrocatalysis.

**Figure 1.**
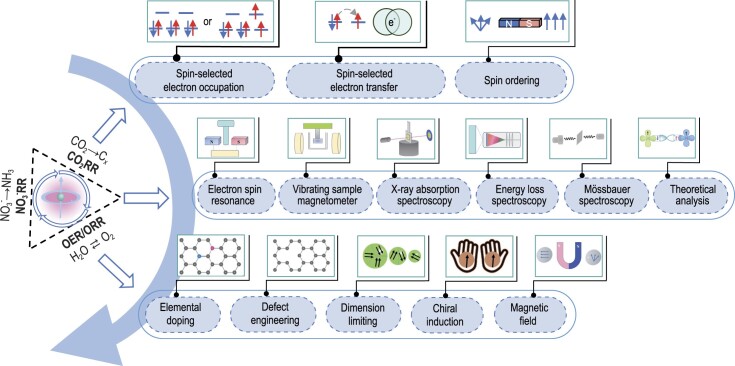
Outline of the spin-dependent electrocatalysis discussed in this review.

The objective of this review is to deliver an up-to-date understanding of spin-dependent electrocatalysis (Fig. 1). The review begins by introducing the fundamental concepts and principles that dominate the spin-related features in electrocatalysts. It then covers state-of-the-art techniques for characterizing spin configuration, alongside the latest theoretical developments. Following this, we proceed to outline the practical strategies for manipulating the spin configuration of catalysts across various electrocatalytic reactions. In the concluding section, we discuss existing challenges and propose some future prospects. This review is expected to provide the electrocatalysis community with a comprehensive overview of spin-dependent electrocatalysis, aiming to address ongoing challenges and exploit forthcoming opportunities in energy conversion.

## SPIN-RELATED FEATURES IN ELECTROCATALYST

Chemical reactions are fundamentally governed by the spin conservation rule, which dictates that the total spin angular momentum of a system remains unchanged unless influenced by external forces [27]. Essentially, this means the overall spin of an isolated system is preserved over time. According to this rule, the coherent combination of two reactants with electrons aligned in parallel to yield a product with a net-zero spin is strictly prohibited [28]. It should be emphasized that the conservation rule applies only to an isolated system or a continuous symmetry. Electrocatalysis offers a platform to overcome it as external voltage is applied and complex spin-related interactions exist. Under electrochemical conditions, the introduction of a suitable paramagnetic electrocatalyst can help provide the necessary energy for a spin flip, or spin selection, enabling the reaction to proceed. The groundbreaking concept of utilizing spin in electrocatalysis was first introduced in the 1980s. It is defined as ‘the phenomenon of promoting a chemical reaction by overcoming spin inhibition or reducing the activation barrier through spin decoupling induced by a paramagnetic catalyst’ [29]. Since its inception, numerous spin-related features have been identified that contribute to the alterations in spin kinetics during electrocatalysis [30]. Therefore, in exploring the intricate mechanisms behind spin-dependent electrocatalysis, a comprehensive understanding of the foundational spin-related features within the electrocatalyst is necessary.

### Spin-selected orbital occupation

**Figure 2.**
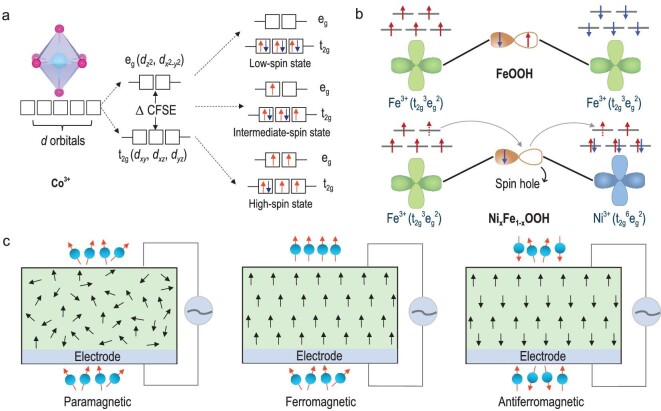
(a) Depiction of how different spin states of octahedral Co^3+^ cation arise from spin-selected orbital occupation. (b) Schematic diagram of spin-selected electron transfer. The top and bottom sections show the electron transfer pathway in FeOOH and Ni*_x_*Fe_1–_*_x_*OOH, respectively. (c) Schematic diagram of electrocatalysts with paramagnetic, ferromagnetic, and antiferromagnetic spin ordering.

In transition-metal–based catalysts, particularly those containing metal cations with *d^n^* (*n* = 4 to 7) electron configurations, the interactions of metal *d* orbitals with distinctive ligand lead to a loss of the quintuple degeneracy, namely ${{d}_{xy}}$, ${{d}_{xz}}$, ${{d}_{yz}}$, ${{d}_{{{z}^2}}}$ and ${{d}_{{{x}^2} - {{y}^2}}}$ [11]. The loss of the quintuple degeneracy leads to a split in their energy levels, giving rise to the formation of t_2__g_ and e_g_ orbitals according to symmetry operations. Taking an octahedral coordinated *d^n^* cation as an example, the *d* orbitals split into three low-lying t_2__g_ orbitals (${{d}_{xy}}$, ${{d}_{xz}}$, and ${{d}_{yz}}$) and two high-lying e_g_ orbitals (${{d}_{{{z}^2}}}$ and ${{d}_{{{x}^2} - {{y}^2}}}$) due to the crystal field. The energy difference between t_2__g_ and e_g_ orbitals is defined as the crystal field splitting energy (CFSE). Following Hund's rule, electrons fill equivalent orbitals in a spin-parallel manner to maximize the system's total spin. When *n* is not larger than 3, all electrons fill the low-lying t_2__g_ orbitals in the same spin direction in compliance with the Pauli exclusion principle. When *n* is larger than 3, if electron pairing energy is lower than the CFSE, the residual electrons (n-3) will initially populate the t_2__g_ orbitals with opposite spin and then occupy the high-lying e_g_ orbital (if *n* is >6), resulting in a low-spin state. Conversely, if the electron pairing energy is higher than the CFSE, electrons will first fill the high-lying e_g_ orbitals before occupying low-lying t_2__g_ orbitals. This results in either an intermediate- or a high-spin state, depending on the specific electronic configuration (Fig. 2a).

It has been suggested that the e_g_ orbitals are particularly compatible in energy and spatially congruent with adjacent O 2*p* orbitals. This characteristic makes electrons in the e_g_ orbitals, rather than those in the t_2__g_ orbitals, more likely to be actively involved with reactants or intermediates in the adsorption processes during electrocatalysis [31,32]. According to Sabatier's Principle (thermodynamic chemisorption principle), the highest reaction rate occurs when the adsorption energy is neither too strong nor too weak [33]. In this regard, the occupancy of the e_g_ orbitals plays a critical role in determining the catalytic activity. Pioneering studies by Shao-Horn and colleagues have elucidated a ‘volcano plot’ relationship between the e_g_ occupancy and the catalytic performance for OER and ORR, suggesting that the number of e_g_ electrons can serve as a powerful descriptor for evaluating oxygen electrocatalysis [34]. The underlying reasons behind the optimal activities observed at an intermediate e_g_ occupancy was further elucidated [11,35].

While utilizing e_g_ occupancy as descriptor offers a straightforward approach to evaluate catalytic performance, strategies of adjusting the e_g_ occupancy of electrocatalysts are yet to be developed [36]. It is well recognized that the modification in the spin states of transition metals of electrocatalysts could result in diverse e_g_ orbital occupancies. Therefore, fine-tuning the spin state of metal cations emerges as a compelling strategy to boost electrocatalytic activity. Zou *et al.* developed an approach, utilizing the secondary coordination sphere effect, which is created by neighboring N=C–N groups, to adjust the spin state of the Fe center in a single atom catalyst from a low-spin to an intermediate-spin state, thereby enhancing ORR activity [37]. Similarly, Xu's team have identified that ZnCo_2_O_4_ catalysts experience a spin state change at varying post-calcination temperatures [38]. Since both the crystal structure and valance state of cobalt cations remain unchanged, the different catalytic performance can be attributed solely to the change in cobalt spin state. These discoveries reveal the pivotal role of spin-selected orbital occupation in spin-dependent electrocatalysis, shedding light on the importance of spin state adjustment in achieving superior catalytic performance.

### Spin-selected electron transfer

The dynamics of electron transfer stands at the core of electrocatalysis, presenting a rich field for exploration. The orientation of electron spins plays a crucial role in facilitating a rapid interfacial electron transfer, enhancing transport to external circuits, and ensuring the availability of active surface sites [39,40]. It's widely acknowledged that good conductivity can mitigate interfacial and bulk transport resistances in electrocatalysts, thereby elevating electrocatalytic efficiency [41,42]. However, improving conductivity alone is not a catch-all solution for achieving substantial increases in catalytic activity. This is particularly noteworthy in OER and ORR, where the majority of electrons share the same spin orientation before being transported. Consequently, traditional conductivity measurements are not reliable in fully capturing the spin-polarized current.

For example, although some oxide or oxyhydroxide catalysts have inferior conductivity compared to noble metals [43], they offer greater regulation freedom in spin manipulation based on the couplings of spins, charges, orbitals, and lattices. When the metal 3*d* orbitals strongly hybridize with the O 2*p* states, metal-oxygen bonds in these catalysts exhibit benign conductivity owing to the mixed ionic-covalent bonding and possible spin hopping (Fig. 2b) [21]. A breakthrough work was reported by Xu's group, who successfully modulated spin-polarized electrons in cobalt-based oxides using a magnetic field [44]. They discovered that the 3*d* orbitals of Co^2+^ in CoFe_2_O_4_ catalysts have more overlapping with O 2*p* orbitals after the spin alignment. This results in an increased hybridization of the 3*d*-2*p* orbitals as well as oxygen ligand holes with a ferromagnetic nature. Consequently, the kinetics of electron transfer at the three-phase interface is optimized by the increased spin density on oxygen atoms.

In contrast, when orbital hybridization is weak, electron transfer is related with the Goodenough-Kanamori rules, such as super-exchange and double-exchange interactions [45,46]. Ding's team provided an insight into the varied OER activities on different metal-doped (Mo, Ru, Fe, Rh, W, Co, Ir) γ-NiOOH [47]. The *in-situ* electrical transport spectroscopy (ETS) measurement indicates that the conductivity of doped NiOOH is correlated with OER activity. The high conductivity of γ-NiOOH doped with group VIII metals is attributed to double-exchange interactions between Ni and Co^4+^/Rh^4+^/Ir^4+^ via the e_g_ and t_2__g_ orbitals, respectively. In contrast, Mo- and W-doped γ-NiOOH exhibit poor conductivity without effective exchange interactions, resulting in reduced catalytic activity. Wu *et al.* further elucidated the role of vibronic super-exchange in the double-encapsulated crystal La_2_NiMnO_6_, which redistributes the *d* electron configuration from a static Mn^4+^–O–Ni^2+^ super-exchange to a dynamic Mn^3+^–O–Ni^3+^ vibronic super-exchange [48]. This modified electron filling state optimizes the surface activation of the catalyst. These findings highlight that the spin-selected electron transfer, both at the surface and within the bulk, plays a crucial role in the reaction dynamics of spin-dependent electrocatalysis.

### Spin ordering

Spin ordering refers to the systematic arrangement of electron spins within a material [49]. Depending on how these spins are aligned relative to each other, different types of spin ordering can be classified (Fig. 2c), including paramagnetic ordering where electron spins are randomly orientated due to thermal motion, ferromagnetic ordering where spins are aligned parallel to each other, antiferromagnetic ordering where adjacent spins point in opposite directions, ferrimagnetic ordering where adjacent spins also point in opposite directions but with unequal spin moment, and non-collinear spin ordering where spins are aligned in more complex patterns that do not fit the simple parallel or antiparallel category.

In spin-dependent electrocatalysis, spin ordering has been demonstrated to have influence on both the thermodynamic reaction pathway and the kinetics of electron transfer, thereby altering the electrocatalytic performance [50,51]. For instance, Sun *et al.* introduced Cu^2+^ into NiFe layered double hydroxides (Cu-NiFe-LDHs), which induced a spin ordering transformation from ferrimagnetic to ferromagnetic [52]. The transformation was caused by the Jahn–Teller distortion of Cu^2+^ with an asymmetrical e_g_ orbital occupancy (t_2g_^6^e_g_^3^) that increased the spin state of the Fe sites. As a result, the as-designed Cu-NiFe-LDHs exhibit an optimized OER free energy pathway than NiFe-LDHs. Recently, Xu's group have constructed a ferromagnetic-paramagnetic interface through a controlled surface reconstruction approach [53]. They employed a spin-pinning effect strategy to align the spin in paramagnetic hydroxyl oxides using a weak magnetic field, which reduces the potential barrier for O–O coupling and enhances the OER activity of Co*_x_*Fe_3−_*_x_*O_4_.

The influence of spin ordering on the kinetics of electron transfer during spin-dependent electrocatalysis mainly originates from the altered spin channel induced by the manipulation of spin ordering [54]. Using the Mn*_x_*V*_y_*O*_z_* family as model electrocatalysts, Ling's team demonstrated that the Mn oxides with ferromagnetic ordering show greatly enhanced manganese (Mn)-oxygen (O) hybridization than those with antiferromagnetic ordering [55]. The enhanced Mn-O hybridization is attributed to the formation of a spin-up channel near the Fermi level by the spin-polarized Mn-3*d* electrons. This accelerates the electron transfer between surface Mn sites and the adsorbed oxygen species, ultimately contributing to the reinforced ORR performance. Xu *et al.* designed an antiferromagnetic inverse spinel LiCoVO_4_ oxide containing high-spin state Co^2+^ cations (t_2g_^5^e_g_^2^) in the octahedral sites [56]. They demonstrated that the as-designed spinel LiCoVO_4_ possesses spin-conductive channels that can enhance the selective removal of spin-orientated electrons from OH^–^ and facilitates triplet oxygen generation during OER. These works further elucidate the important role of spin ordering in electron transfer in spin-dependent electrocatalysis.

## CHARACTERIZATION TECHNIQUES AND THEORETICAL ANALYSIS

To fully elucidate the mechanism of spin-dependent electrocatalysis, it is essential to establish a connection between the spin configurations of electrocatalysts and their catalytic performance. This requires the ability to visualize both short-range and long-range spin configurations, which necessitates proficiency in characterization techniques and theoretical analysis. In this section, the characterization of electron spin resonance, including vibrating sample magnetometer, X-ray absorption spectroscopy, electron energy loss spectroscopy, and Mössbauer spectroscopy, as well as theoretical analysis approaches, are introduced. It should be noted that, in most cases, the above-mentioned techniques are employed simultaneously to cross-validate the obtained spin configuration of electrocatalysts.

### Electron spin resonance

Electron spin resonance (ESR), also known as electron paramagnetic resonance (EPR), is a powerful tool to characterize the magnetic and electronic properties of materials with unpaired electrons [57]. It operates based on the principle that electrons with unpaired spins in an external magnetic field can absorb microwave radiation at a frequency that matches the energy gap between two spin states, causing a transition from a lower spin state to a relatively higher one. The absorption can be recorded as an ESR spectrum, the position, intensity, and shape of which provide information that can be derived to picture the spin configuration of electrocatalysts, including the number of unpaired electrons, the orbital occupancy of certain metal cations, and the local magnetic field.

**Figure 3.**
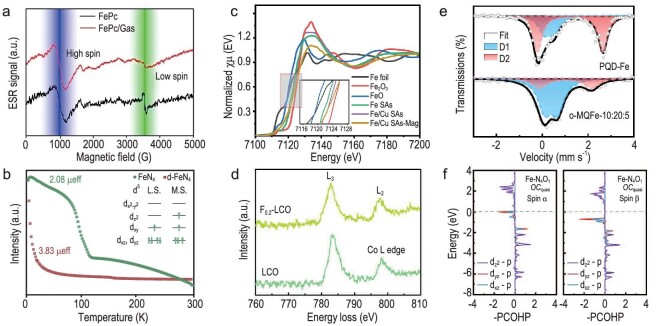
(a) ESR spectra of FePc and FePc incorporated with GaS (FePc/GaS) recorded at 100 K. Blue and green areas identify the range of high- and low-spin signals, respectively. Reproduced with permission from Ref. [59]. Copyright 2022, Wiley-VCH GmbH. (b) Magnetic susceptibility calculated by *M*–*T* curve for d-FeN_4_ and FeN_4_. The upper right inset shows the occupation of d-orbital electrons in both samples. Reprinted with permission from Ref. [61]. (c) Fe K-edge XANES spectra of the Fe SAs, Fe/Cu SAs and Fe/Cu SAs-Mag. Reproduced with permission from Ref. [67]. Copyright 2023, Wiley-VCH GmbH. (d) The EELS spectrum of Co L-edge for LaCoO_3_ (LCO) and F-doped LaCoO_3_ (F_0.2_-LCO). Reprinted with permission from Ref. [69]. Copyright 2022, Wiley‐VCH GmbH. (e) ^57^Fe Mössbauer spectra of Fe-chelated polymer-like quantum dots (PQD-Fe) and O-terminated MXene nanosheet (o-MQFe). Reprinted with permission from Ref. [70]. Copyright 2022, Wiley-VCH GmbH. (f) The crystal orbital Hamilton populations (COHP) analysis derived from density of states (DOS) of Fe–O bond for Fe-N_4_ of Fe-N_4_O_1_ with quasi-octahedral coordination (Fe-N_4_O_1_  *OC*_quasi_) after *OH adsorption. Reprinted with permission from Ref. [71]. Copyright 2023, Wiley-VCH GmbH.

In fact, ESR has been intensively employed in the investigation of spin-dependent electrocatalysis. For example, Xie's team prepared Co_3_S_4_ atomically thin nanosheets (CSATN) via an ultrasound exfoliation treatment, which exhibits much higher activity towards OER compared to bulk Co_3_S_4_ [58]. The superior activity was found to originate from the spin state transformation of cobalt from low- to high-spin state on CSATN, which was revealed via ESR measurement. Li and coworkers incorporated an *n*-type gallium mono-sulfide (GaS) with *p*-type iron phthalocyanine (FePc) to fabricate *p*-*n* junctions, which realized a ∼2.5-fold increase in ORR activity on the active FeN_4_ moiety [59]. They applied ESR characterization to reveal an intermediate- to high-spin state transition in the Fe^2+^ center as a result of the *p*-*n* junction fabrication (Fig. 3a). This spin transition makes FeN_4_ moiety more readily adsorb and dissociate O_2_ molecules, thereby enhancing the ORR performance.

### Vibrating sample magnetometer

Vibrating sample magnetometer (VSM) is effective in measuring the magnetic moment of a sample, thereby giving information of the spin configuration such as spin state and orbital occupancy. It operates by applying a magnetic field to a sample and measuring the resulting magnetization [60]. During measurement, the sample is placed in a magnetic field, which induces a magnetization within the sample. When the magnetized sample is vibrated, it introduces a voltage proportional to the sample's magnetic moment. A set of coils can be arranged therein to measure the induced voltage to obtain the magnetization plot as a function of the applied magnetic field and temperature.

Based on the Curie-Weiss law, the measured magnetization intensity versus temperature (*M*–*T*) curve by VSM can be utilized to derive the effective magnetic moment (*μ_eff_*) of the sample, thereby inferring the spin state of metal cations. For instance, by measuring the *M*–*T* curves of dangling-FeN_4_ (d-FeN_4_) and FeN_4_ centers, Wang's team calculated the *μ_eff_* value of dangling-FeN_4_ (d-FeN_4_) to be 3.83 *μ_B_*. According to the formula $\ {{\mu }_{eff}} = \sqrt {n*( {n + 2} )} $ (*n* is the number of unpaired *d* electrons), the number of unpaired *d* electrons of d-FeN_4_ is inferred to be 3. This indicates three unpaired electrons filling in ${{d}_{{{z}^2}}}$, ${{d}_{xy}}$, and ${{d}_{xz}}$, ${{d}_{yz}}$ orbitals (Fig. 3b), demonstrating an intermediate-spin state of d-FeN_4_ [61]. Taking advantage of *M*–*T* curves, Liang's research group identified the *μ_eff_* values of spinel Ni*_x_*Co_3−_*_x_*O_4_ with different Ni ratios, based on which the e_g_ occupancy of nickel cations were inferred [62]. The established electrocatalytic activity of urea oxidation reaction (UOR) as a function of the e_g_ filling suggests that a moderate e_g_ occupancy is advantageous for achieving high UOR activity. Employing VSM characterization, Xia *et al.* obtained a *μ_eff_* of 0.588 μ_B_ for the Ru cations in *P*_21_/*m*-Li_2_RuO_3_, corresponding to a low-spin state (Ru^4+^, t_2g_^4^e_g_^0^). In contrast, the *μ_eff_* was 2.49 μ_B_ for Ru cations in *C*_2_/*c*-Li_2_RuO_3_, which can be assigned to the high-spin state (Ru^4+^, t_2g_^3^e_g_^1^) [63]. Since the interaction between the central metal and the ligand oxygen is closely related to the spin configuration of metal cations, the reduced oxidation potential of anion redox in *C*_2_/*c*-Li_2_RuO_3_ can be attributed to the high-spin state Ru^4+^.

### X-ray absorption spectroscopy

X-ray absorption spectroscopy (XAS), particularly through its X-ray absorption near edge structure (XANES) region, is also a powerful tool to deduce the spin state of metal cations. This is achieved by examining the absorption edge and related features tied to the electronic configuration of the metal cations [64]. The shifts in edge position and variations in the shape and intensity of the XANES spectra are indicative of the oxidation state and coordination environment, which can, in turn, be used to infer the orbital occupancy and spin state of metal cations.

The employment of XANES to identify the spin configurations of electrocatalysts has been intensively reported in electrocatalysis. By analyzing the XANES spectra of LaCoO_3_ (LCO) epitaxial thin films with varying crystallographic orientations, Wu's team uncovered a spin state transition from low-spin state (LCO(111) film) to intermediate-spin state (LCO(100) film) [65]. The transition to a higher spin state increases the occupancy of e_g_ orbitals, which in turn enhances the OER activity by optimizing the adsorption free energy of reaction intermediates. Hou and co-workers utilized the XANES spectra of Fe K-edge to reveal a spin state transition of Fe centers in a single-atom catalyst (SAC) induced by sulfur doping [66]. The introduction of S into the Fe single atom catalyst can effectively modulate the high-spin state Fe center to an intermediate-spin state, facilitating the electrocatalytic activity of nitrogen reduction reaction (NRR) via the activation of N≡N triple bond. On the contrary, Fu *et al.* found that the high-spin state Fe centers in an Fe single-atom catalyst, compared to low- and intermediate-spin state centers, is advantageous in catalyzing ORR [67]. The Fe K-edge XANES spectra characterized a t_2g_^3^e_g_^2^ spin configuration of Fe^3+^ center in a magnetic-field–treated Fe/Cu single-atom catalyst (Fe/Cu SAs-Mag), which is further demonstrated to facilitate ORR through the optimization of O_2_ adsorption and the promotion of the rate-determining step (from *O_2_ to *OOH) (Fig. 3c).

### Electron energy loss spectroscopy

Electron energy loss spectroscopy (EELS) is a powerful analytical technique used in transmission electron microscopy (TEM) to analyze the energy loss of electrons as they interact with a sample [72]. It provides valuable information about the elemental composition, chemical bonding, and electronic structure of materials at the nanoscale. EELS can also be used to detect the spin state of materials by examining the fine structure of energy losses, particularly near the core loss edges like the L-edge for transition metals [68]. The analysis of these loss features, including peak splitting and intensity variations, reflects changes in the electronic structure that are indicative of different spin states. By comparing the observed spectral characteristics with theoretical models or known standards, EELS enables the precise determination of the spin states in a wide range of materials, from magnetic compounds to complex oxides.

By analyzing the EELS spectra of the bulk and nanosized LaCoO_3_ (LCO) at Co L-edge and O K-edge, Zhou and co-workers identified a weakened Co 3*d*-O 2*p* hybridization at nanosized LCO, based on which a spin state transition of Co^3+^ from low-spin state to high-spin state can be deduced [73]. The improved OER activity on nanosized LCO can therefore be attributed to the high-spin state Co^3+^ with optimized e_g_ occupancy. Similarly, Gao *et al.* doped fluorine into the lattice of LaCoO_3_ and observed the spin state of Co^3+^ changing from low-spin state to intermediate-spin state via the Co L-edge EELS spectra (Fig. 3d) [69]. The spin state transition of Co^3+^ results in enhanced electrocatalytic activity for both OER and ORR. Qiao's team synthesized a spinel Co_2_VO_4_ catalyst marrying metallic vanadium atomic chains with electroactive cobalt cations for highly efficient and stable ORR [74]. The EELS analysis helps identify a low-spin state Co^2+^ in the octahedral sites, which enhances the catalytic activity via providing an optimized e_g_ filling and increases the electroconductivity via promoting the formation of V^4+^-V^4+^ octahedral units.

### Mössbauer spectroscopy

Mössbauer spectroscopy is a nuclear gamma-ray resonance technique that provides detailed information of the atomic-scale properties of materials, such as their electronic, structural, and magnetic characteristics [75]. It is highly sensitive to the hyperfine interactions between nuclear states and the electronic environment, enabling the detection of subtle changes in electron density, oxidation states, and spin ordering. By analyzing the hyperfine splitting in the Mössbauer spectrum, which arises from the interaction of the nuclear magnetic moment with the local magnetic field, this technique can precisely identify the spin state and magnetic features of materials, offering invaluable information to visualize both short-range and long-range spin configurations.

For spin-dependent electrocatalysis, Mössbauer spectroscopy can be utilized to identify the oxidation state and spin-selected orbital occupancy of metal cations. Zhai's team employed both *ex-situ* and *in-situ*  ^57^Fe Mössbauer spectroscopy and discovered that introducing sulfur (1.3 wt%) into an Fe single atom catalyst can lead to the formation of a significant proportion (58%) of low-spin state Fe^3+^ centers [76]. By adjusting the amount of doped sulfur in an Fe single atom catalyst, a clear correlation emerges between the ORR performance and the proportion of low-spin state Fe^3+^ centers, which demonstrates the responsibility of low-spin state Fe^3+^ for enhanced catalytic activity. Moreover, Wang *et al.* revealed that the D1 and D2 peaks in the Mössbauer spectrum of Fe-chelated polymer-like quantum dots (PQD-Fe) are indicative of the intermediate- and low-spin state of Fe^3+^, respectively (Fig. 3e) [70]. Furthermore, the area represented by the D1 peak in PQD-Fe (34.6%) is smaller than that in O-terminated MXene nanosheet (o-MQFe) (72.6%), confirming a spin sate transition of Fe^3+^ from low- to intermediate-spin state.

### Theoretical analysis

Traditional experimental characterization techniques are powerful in visualizing the spin configuration of electrocatalysts; however, patterning the whole reaction mechanism necessitates the establishment of a bridge between theoretical concepts and experimental observations. This calls for the employment of theoretical analysis to delve deeper into the electronic structures, orbital interactions, and spin-sensitive pathways in electrocatalysis. In recent years, various theoretical analysis approaches have been developed to elucidate the experimentally observed phenomena, which, in turn, predict the performance of newly engineered electrocatalysts.

A common approach of theoretical analysis is the employment of quantum computations, including the density functional theory (DFT) calculations of the density of states (DOS), charge distribution, spin density, orbital mapping, and free energy pathways. Wang *et al.* introduced an innovative method to adjust the splitting of Fe *d* orbitals at the FeN_4_ site by introducing axial coordination. They calculated the DOS, spin density, and ORR free energy pathways of the as-designed single atom electrocatalyst. Their calculation result indicates that axial interactions can alter the local crystal field from square-plane to a quasi-octahedral coordination, triggering a spin configuration shift from intermediate- to low-spin state. This shift reduces the number of unpaired electrons in the ${{d}_{{{z}^2}}}$, ${{d}_{xz}}$, and ${{d}_{yz}}$ orbitals. As a result, the adsorption/desorption energies of intermediates are adjusted to moderate, facilitating a four-electron oxygen reduction process (Fig. 3f) [71].

Another approach, which has emerged recently, is the incorporation of conceptual analysis to describe spin-dependent electrocatalysis. Methodologies within this approach include the employment of exchange interactions, atomic orbital interactions, and charge transfer pathway to elucidate the reaction performance. Xu's group proposed a strategy of analyzing the orbital interactions between the metal center and key reaction intermediates to identify the active cation in Ni*_x_*Fe_1−_*_x_*OOH, which highlights the superior activity of an iron cation over a nickel cation [21]. By adopting the insights of super-exchange interactions, Shao and his team predicted perovskite SrTi_0.7_Ru_0.3_O_3-_*_δ_* (STRO) oxide as a highly active single-phase hydrogen evolution catalyst, justified by excellent activity in alkaline media due to charge redistribution of the Ti^3+^–O–Ru^5+^ super-exchange units [77].

## STRATEGIES TO MANIPULATE SPIN CONFIGURATIONS

To unlock the full potential of spin-dependent electrocatalysis, it is important to develop effective strategies that enable the manipulation of the spin configurations of electrocatalysts. In recent years, a variety of strategies have been developed, including but not limited to elemental doping [78], defect engineering [79], dimension limiting [80], chiral induction [81], and magnetic field manipulation [44]. These approaches offer the possibility to precisely control the spin configurations of electrocatalysts, thereby significantly impacting their electrocatalytic performance. Herein, we introduce the foundational theories underpinning these approaches and spotlight their recent advances in spin-dependent electrocatalytic applications such as OER, ORR, NRR, and CO_2_ reduction. This will showcase the significant influence of these approaches in modifying electrocatalytic performance, thereby offering insightful perspectives into the future of energy conversion technologies.

### Elemental doping

Elemental doping, which refers to the introduction of foreign atoms into the lattice of host materials, is widely employed as a strategy capable of altering the spin configuration of electrocatalysts. This alteration arises from the fact that the dopant atoms can have different electronegativities or orbital occupancies compared to the host atoms, affecting the distribution and alignment of electron spins. The changed spin configuration may adjust the adsorption/desorption energy of reaction intermediate, influence the electron transfer route, modify atomic exchange interactions, and thereby optimize reaction performance [78,82].

**Figure 4.**
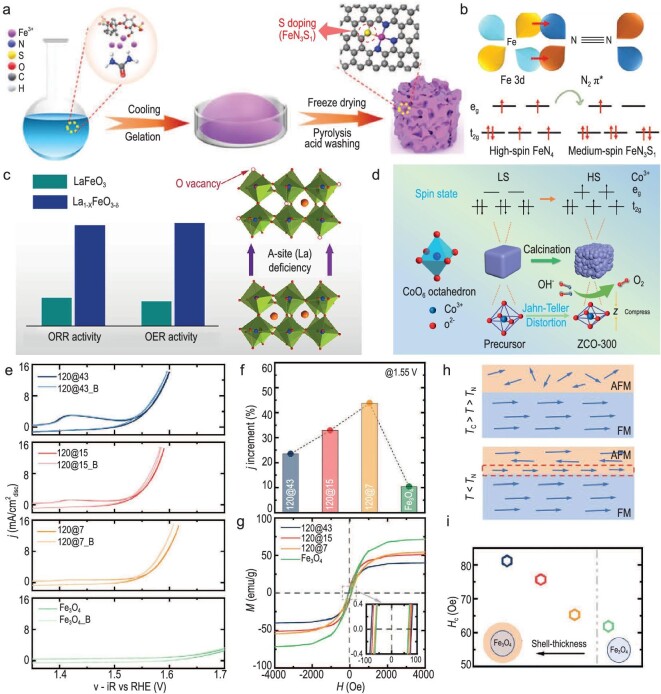
(a) Schematic diagram of the synthesis procedure of Fe single atoms with sulfur ligand doping (FeN_3_S_1_). (b) Molecular orbital diagram of N_2_ and possible Fe spin configurations in FeN_4_ and FeN_3_S_1_. Reprinted with permission from ref. [66]. Copyright 2023, Wiley-VCH GmbH. (c) Schematic illustration of the enhanced OER/ORR activity on A-site cation deficient LaCoO_3_ with oxygen vacancies (La_1−_*_x_*FeO_3−_*_δ_*). Reprinted with permission from Ref. [88]. Copyright 2016, American Chemical Society. (d) Schematic illustration of the spin state change of cobalt cations in spinel ZnCo_2_O_4_ with the introduction of lattice distortion. Reprinted with permission from Ref. [90]. Copyright 2023, American Chemical Society. (e) OER performance of 120 nm Fe_3_O_4_ NPs with different shell thicknesses of 43 nm, 15 nm, 7 nm, and 0 nm with or without a magnetic field. (f) Current density *j* increment (%) at a potential of 1.55 V vs. RHE with a magnetic field related to that without a magnetic field. (g) Hysteresis loops (MH curve) for 120 nm Fe_3_O_4_ NPs with different shell thicknesses of 43 nm, 15 nm, 7 nm, and 0 nm. The inset shows the enlarged area of *H_c_* in the gray dashed box. (h) Schematic illustration of FM-AFM coupling. (i) *H_c_* values of 120 nm Fe_3_O_4_ NPs with different shell thicknesses of 43 nm, 15 nm, 7 nm, and 0 nm under a magnetic field. Reprinted with permission from Ref. [39]. Copyright 2021, Wiley‐VCH GmbH.

The employment of elemental doping to fine-tune the spin configuration of electrocatalysts has been intensively reported. For instance, Shao *et al.* reported a spin-polarization strategy that creates a net spin moment by doping manganese into the lattice of antiferromagnetic RuO_2_ [83]. This study shows that Mn doping induces changes in the magnetic structure of RuO_2_. Specifically, the interaction between RuO_2_ lattice and Mn^2+(3+)^ ions produces a long-range ferromagnetic ordering, which increases the magnetic moments on Ru atoms. The *d*-electrons in Ru atoms undergo spin alignment under an external magnetic field, creating a new spin-polarized environment for OER. This doping-induced spin tuning dramatically improves the OER activity and enhances the stability of catalysts. Liu and colleagues successfully introduced Fe^3+^ ions into spinel Co_3_O_4_, which results in a delocalization of Co 3*d* electrons and induces a spin state transition of Co^3+^ from low-spin state to intermediate-spin state [84]. Their study demonstrates that Fe^3+^ cations are effective in activating adjacent Co^3+^ cations through spin and charge effects. Hou's group has reported a feasible approach for modulating the spin state of Fe single atoms through ligand doping with sulfur (FeN_3_S_1_) (Fig. 4a) [66]. The introduction of sulfur into isolated Fe-N moieties gives rise to a spin state change of Fe center from a high-spin state to an intermediate-spin state (Fig. 4b). A lowered free energy barrier for *NHH intermediate formation and accelerated hydrogenation reaction kinetics on the isolated FeN_3_S_1_ site are revealed by theoretical calculations, which facilitates the electrochemical reduction of N_2_ to NH_3_.

### Defect engineering

Defect engineering is the deliberate introduction or manipulation of defects within the lattice of materials to tailor their properties for specific applications. These defects can include vacancies (missing atoms), interstitials (extra atoms positioned between the lattice atoms), lattice distortions (disruptions in the orderly sequence of the lattice), and grain boundaries (interfaces where crystal orientations change) [85,86]. Certain types of defects have been demonstrated to be capable of altering the localized spin orientation and spin density, thereby influencing spin configuration. Moreover, subtle manipulation of certain defects can also fine-tune the atomic exchange interactions within the lattice of an electrocatalyst, creating a new pathway to promote spin-selected electron transfer [87]. Therefore, rational utilization of defect engineering can yield remarkable enhancement in spin-dependent electrocatalysis.

A commonly employed strategy to regulate defects for adjusting the spin configuration of electrocatalyst is vacancy engineering. Jiang *et al.* used MoS_2_ with sulfur vacancy (S-vacancy MoS_2_) as a substrate to capture a single transition-metal (TM) atom onto its basal plane [79]. DFT calculations revealed that the non-covalent interactions between S-vacancy MoS_2_ and TM can induce strong spin polarization on the exposed Mo atoms, thereby promoting the adsorption of N_2_ and its subsequent hydrogenation to NH_3_. Shao's team introduced an oxygen vacancy into the lattice of A-site deficient perovskite LaFeO_3_. They discovered that oxygen vacancies in the A-site defective lattice can enhance both O_2_ chemisorption and spin-selective electron transport [88]. At the same time, the A-site cation deficiency and oxygen vacancies can induce the presence of Fe^4+^ species in the spin configuration of t_2g_^3^e_g_^1^. Compared to pristine LaFeO_3_, the vacancy-engineered La_1−_*_x_*FeO_3−_*_δ_* (*x* = 0.05) exhibits superior electrocatalytic activity for both OER and ORR (Fig. 4c).

Other types of defects, such as interstitials, lattice distortions, and grain boundaries, have also been developed to engineer the spin configuration of electrocatalysts. Zhao and coworkers designed a boron-interstitial C_2_N catalyst and investigated its electrocatalytic activity towards nitrogen fixation and reduction [89]. The as-designed catalyst exhibits a spin-polarized feature on boron site, which can effectively capture and activate N_2_ molecules via an ‘acceptance-donation’ process, demonstrating remarkable activity for NRR. Li's group developed a strategy of temperature-induced lattice distortion to manipulate the spin state of cobalt cations in spinel ZnCo_2_O_4_ [90]. They demonstrated that the calcination temperature can precisely control the distortion of the CoO_6_ octahedra units and thus manipulate the spin state of Co^3+^ cations. The study revealed that ZnCo_2_O_4_ calcinated at 300°C (ZCO-300), which consists of Co^3+^ cations with 64.4% in high-spin state and 35.6% in low-spin state, possesses the most favorable spin configuration for catalyzing OER (Fig. 4d). Su and his team incorporated grain boundary into hexagonal boron nitride (GB-h-BN), which was thereafter utilized as a support to anchor single-atom Mo (Mo@GB-h-BN) [91]. Compared to pristine h-BN, GB-h-BN can facilitate the generation of low-spin state Mo with enhanced *NH_3_ binding affinity. Consequently, Mo@GB-h-BN demonstrates greatly improved activity for catalyzing NRR.

### Dimension limiting

Dimension limiting, which refers to the process of reducing the dimensions of a material (e.g. from bulk to two-dimensional or even one-dimensional structures), is a robust strategy to alter the spin configuration of electrocatalysts through enhanced surface effects and quantum confinement. When materials are constrained to lower dimensions, the proportion of atoms that located at the surface region increases dramatically, which greatly amplifies the influence of surface energy on the properties of materials [80]. Due to reduced coordination numbers and distorted atomic orbitals, these surface atoms often exhibit different electronic environments compared to their bulk counterparts. This can affect the distribution and alignment of unpaired electron spins, thus modifying the spin configuration of electrocatalysts.

Besides, quantum confinement in reduced dimensions can also change the electronic structure of materials. As the motion of electrons becomes restricted to fewer dimensions, the gap between energy levels can change, which could potentially alter the orbital occupancy of electrons, thereby influencing the spin state. These modifications in energy levels and spin state directly impact the spin configuration of electrocatalysts and, consequently, alter their electrocatalytic activity [92]. Hence, dimension limiting could serve as a powerful approach to tailor the spin configurations and catalytic properties of electrocatalysts for specific reactions.

The strategy of dimension limiting has been intensively employed in spin-dependent electrocatalysis. For example, by reducing the particle size of perovskite LaCoO_3_ to ∼80 nm, Zeng *et al.* successfully increased the e_g_ filling of cobalt cations from unity to a value close to the optimum 1.2 [73]. As a result, the best OER activity is achieved in the sample with e_g_ number equaling 1.2. The increment of e_g_ number from 1 to 1.2 is attributed to the spin state transition from low-spin to high-spin state for cobalt cations at the surface of the nanoparticles. Combining theoretical calculations and experiments, Yan's team demonstrated that Rh particles that confined to nanoscale dimensions (∼2.5 nm) have large magnetic moments and exhibit a high-spin state configuration [93]. Even after being anchored to graphene, these Rh nanoparticles still retained their high-spin state configuration, which promotes the adsorption of N_2_ and thereby activates the subsequent hydrogenation to NH_3_.

Xu's group designed Fe_3_O_4_@Ni(OH)_2_ core-shell catalysts with ferromagnetic (FM)-antiferromagnetic (AFM) spin ordering [39]. This interface coupling facilitates selective removal of electrons with spin direction opposite to the magnetic moment of the FM core, thereby improving the OER kinetics of electrocatalysts. The thickness of the AFM shell layer is key in maintaining the coupling effect and enhancing OER activity. They further revealed that the OER activity of the as-prepared samples can be enhanced by an applied magnetic field, with the enhancement increasing as the shell layer thickness decreases. In addition, the magnetic domain in the FM core structure was found to play an important role in influencing electron transport. In a multi-domain FM core, the applied magnetic field can align the magnetic domains, thus optimizing electron transport (Fig. 4e–i). In a single-domain FM core with ordered magnetic dipoles, the spin-selective electron transport can be promoted even without an applied magnetic field.

### Chiral induction

Chiral induction refers to a strategy that utilizes a chiral molecule, a molecule that has the same composition and conformation as its non-superimposable mirror image, to influence the stereochemical outcome of a reaction. Chiral induction has been developed to tune the spin-dependent electrocatalysis via the effect of chiral-induced spin selectivity (CISS) [81]. The CISS effect refers to a phenomenon where the transmission of electrons with a certain spin orientation is preferentially allowed to pass through the chiral molecules. It implies that chiral molecules can act as spin filters, allowing electrons with one specific spin orientation to pass through more readily than those with the opposite spin orientation [94]. The CISS effect bridges the fields of spintronics and chirality, offering potential applications in developing novel catalysts for spin-dependent electrocatalysis.

**Figure 5.**
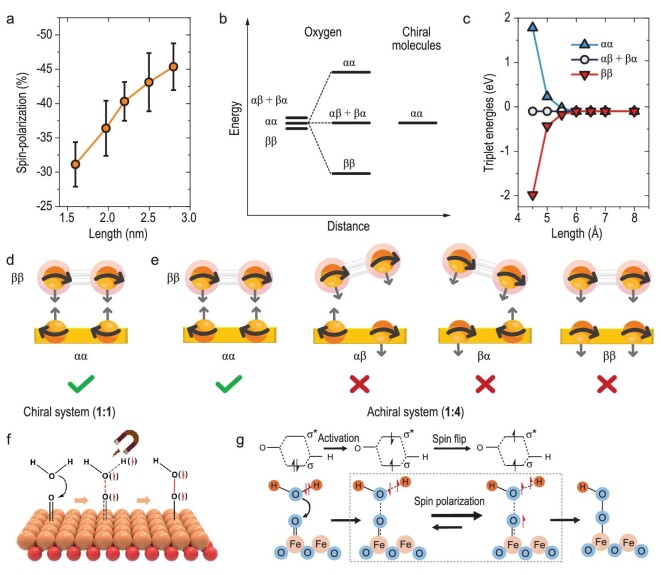
(a) Correlation of the spin polarization with the length of chiral oligopeptides. (b) Spin state splitting in the interaction of the triplet oxygen with the spin-polarized electrons residing on the chiral molecules. (c) The triplet O_2_ energy level as a function of the distance between chiral molecule and oxygen atom. The possible spin states in the case of (d) a chiral system and (e) an achiral one. Reprinted with permission from Ref. [95]. Copyright 2022, National Academy of Science. (f, g) OER mechanisms with spin polarization for coupled O–O bonding and O–H breakage. Reprinted with permission from Ref. [97]. Copyright 2023, Wiley-VCH GmbH.

Recently, the strategy of chiral induction emerged as a means to boost electrocatalytic activity by utilizing the distinctive influence of chirality on electron spin. Naaman *et al.* revealed that chiral electrodes (composed by gold film coated with enantiopure chiral cysteine and oligopeptides) exhibit much higher current densities and lower overpotential than non-chiral ones (consisting of gold film coated with non-chiral alkanethiol molecules) in catalyzing ORR (Fig. 5a–e) [95]. The phenomenon is attributed to the CISS effect on electron current by the chiral molecules. Suda and co-workers designed a chiral TiS_2_ catalyst via electrochemical intercalation of chiral molecules into a metallic TiS_2_ single crystal [43]. The chiral TiS_2_ electrode exhibits more enhanced OER activity than its non-chiral counterpart, which is also attributed to the remarkable CISS effect for triplet oxygen generation.

Waldeck's group explored the key role of spin polarization in promoting OER, which is generated through CISS effects. Their study demonstrates that chiral Co_3−_*_x_*Fe*_x_*O_4_ nanoparticle electrocatalysts are more effective in reducing the overpotential for OER compared to non-chiral catalysts [96]. They found that the rate-determining step (RDS) of the designed chiral catalysts has been altered by the CISS effect. Besides, the chiral catalysts can also polarize the spin of surface intermediates to promote the generation of oxygen in the triplet state. Consequently, it was concluded that spin alignment on the surface of chiral electrocatalysts is a feasible strategy that can be utilized to improve OER efficiency.

### Magnetic field manipulation

Integrating an external magnetic field with electrochemical reactions is a novel strategy that developed recently to enhance spin-dependent electrocatalysis [98,99]. Most electrocatalysts exhibit paramagnetic features under ambient conditions. In other words, in the absence of an external magnetic field, the unpaired electron spins within the paramagnetic electrocatalysts are usually randomly orientated, resulting in a net moment of zero. Meanwhile, some catalysts may be ferro-/ferri-magnetic, however, their macroscopic total magnetic moment may also be zero due to the inconsistent orientation of magnetic domains. When an external magnetic field is applied, the magnetic moments/domains will experience a force that can align them in the direction of the external field [100]. This alignment can lead to a net magnetization of the electrocatalyst in the direction of the applied field, reducing its magnetic entropy.

The strategy of magnetic field manipulation has been widely employed in spin-dependent electrocatalysis, particularly in OER where the generation of triplet oxygen is highly spin-sensitive. Galán-Mascarós and his collaborators observed a direct OER enhancement of magnetic electrocatalysts under a moderate magnetic field (≤450 mT) [101]. The enhancement was found to show a dependence on the bulk magnetization of electrocatalyst, with higher bulk magnetization exhibiting more obvious enhancement. Xu's group have uncovered the underlying mechanism of magnetic-field–enhanced OER performance on magnetic oxides [44]. Utilizing ferromagnetic CoFe_2_O_4_ under a constant magnetic field as the spin polarizer for spin selection, they found a significant OER enhancement. The spin polarization was demonstrated to occur at the first electron transfer step during OER with fast kinetics, where there is a coherent spin exchange between the ferromagnetic catalyst and the adsorbed oxygen species under the principle of spin angular momentum conservation. In contrast, such a phenomenon was not observed in non-ferromagnetic catalysts. This work showcases the way in which magnetic field enhances the spin-polarized kinetics of OER, providing valuable insights into the understanding and utilization of magnetic fields in spin-dependent electrocatalysis. Following this, Sheng *et al.* identified that ferrimagnetic Fe_3_O_4_ exhibits a much higher OER increment in weak alkaline electrolyte (pH 9) than in strong alkaline (pH 14) under an external magnetic field [97], which can be attributed to the formation of spin-polarized intermediates via the nucleophilic attack of Fe^4+^=O by H_2_O in a weak alkaline environment (Fig. 5f, g).

In addition to OER, some other electrocatalytic reactions have also been demonstrated to benefit from the strategy of magnetic field manipulation. Yao's team prepared an oxide-derived copper (OD-Cu) catalyst with surface copper site carrying magnetic moments [60]. Compared to the non-magnetic Cu catalyst, the OD-Cu catalyst exhibits much higher current density and product selectivity towards C_2_ product in CO_2_ electrochemical reduction under the magnetic field. The phenomenon is primarily attributed to the spin-antiparallel alignment of electrons induced by the applied magnetic field, which promotes C–C coupling on the magnetic copper sites. Wang and coworkers synthesized ferromagnetic bowl-like MoS_2_ flakes via the chemical vapor deposition method [102]. They found that the hydrogen evolution activity of the as-prepared MoS_2_ electrocatalyst shows significant improvement when measured under an external vertical magnetic field. The improvement is credited to the capability of ferromagnetic MoS_2_ flakes to efficiently facilitate electron transfer from a glassy carbon electrode to the surface-active sites when subjected to a magnetic field.

It is worth noting that many catalysts can give current increase upon applying an external magnetic field. However, the increment given by most of them is not related to the spin effect. For example, the gas bubble generated in water splitting reactions (including OER and HER) at high current density can block the electrode surface from the electrolyte. As a result, the current density is lower than expected. The gas bubble release can be promoted by applying a magnetic field to the working electrode. The current density will be improved accordingly. This is mainly due to the magnetohydrodynamic (MHD) convection effect. A more detailed discussion about other effects given by an external magnetic field, which are not related to the spin effect, can be found in our earlier paper [103].

## CONCLUSIONS AND PERSPECTIVES

**Figure 6.**
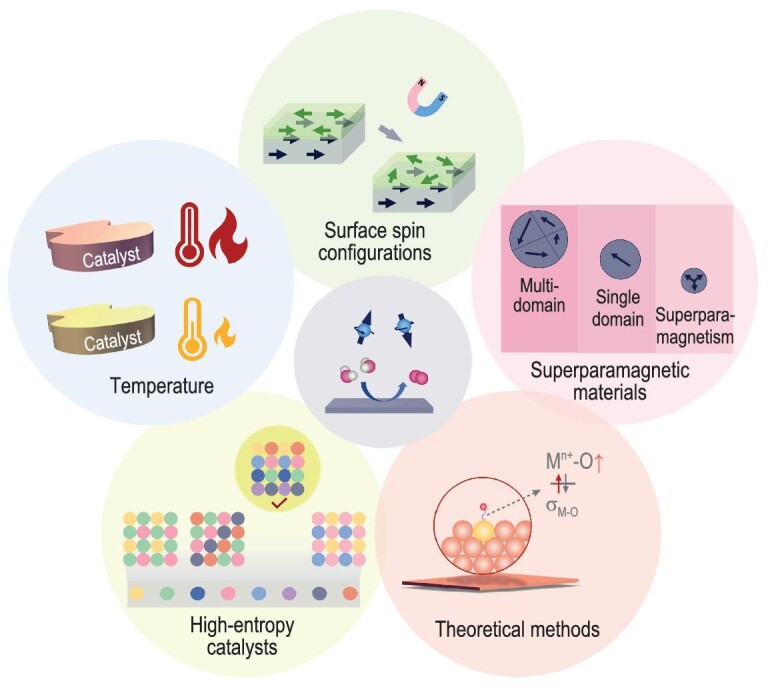
Illustration of future research perspectives in spin-dependent electrocatalysis. Attention can be given to the influence of temperature, the effect of surface spin configuration, superparamagnetic materials, high-entropy catalysts, and the development of theoretical methods.

The exploration of spin-dependent electrocatalysis for enhanced energy conversion efficiency is attracting growing interest. Over the years, a comprehensive set of methodologies has been developed to enable the visualization and adjustment of the spin configuration within electrocatalysts, based on which spin-catalyzed reaction mechanism can be patterned and utilized. This review highlights the key spin-related features of electrocatalysts, the characterization techniques to identify spin configurations, as well as the strategies capable of fine-tuning these configurations. Despite these advancements, the complex nature of spin-dependent electrocatalysis still presents numerous challenges in the accurate design of highly efficient catalysts. In this context, we will briefly discuss several potential prospects for future research (Fig. 6):

1. To identify the influence of temperature on spin-dependent electrocatalysis. In practical scenarios, electrocatalysis typically occurs at a temperature higher than ambient conditions. For example, the operating temperature of water electrolysis in industry is from 70 to 90°C. However, most ferromagnetic catalysts possess Curie temperatures near and even lower than ambient temperature. When the working condition is higher than the Curie temperature, their magnetic structure will transition to a paramagnetic state, thereby weakening the capability of spin-modulated catalytic performance. Moreover, some specific spin states of metal cations are exclusively achievable at low temperature conditions. This is different from the operating temperatures which should be taken into consideration. Therefore, understanding the effects of temperature on spin configuration holds significance for practical spin-dependent electrocatalysis applications.
2. To obtain the surface spin configuration and establish its relationship with electrocatalytic activity. Currently, most electrocatalysts are synthesized as nanostructured materials or polycrystalline powders. These materials often possess surfaces with undefined morphologies, areas, and defects. Such surface characteristics can result in spin configurations that greatly differ from those in bulk. Given that reactions primarily take place on the surfaces of electrocatalysts, accurately identifying the surface spin configuration becomes imperative for establishing a reliable relationship between spin configuration and the intrinsic electrocatalytic activity. Consequently, advancing characterization techniques that are capable of precisely revealing the spin configuration on the surface region is vital for establishing these relationships.
3. To explore spin-dependent electrocatalysis on superparamagnetic materials. Current research on the magnetic-field–enhanced electrocatalysis predominantly focuses on ferromagnetic electrocatalysts with multiple domains, leaving the investigations on those with other types of spin ordering unexplored. For instance, superparamagnetic materials (single domain), similar to the ferromagnetic ones, also exhibit strong magnetization in the presence of an external magnetic field. Excitingly, unlike ferromagnetic materials, superparamagnetic materials do not retain any magnetization once the external field is removed due to thermal perturbation. It has advantages in rapid response to external magnetic field and swift recovery upon its removal, which enables practical benefits to gain activity enhancement and catalyst reutilization. Therefore, superparamagnetic electrocatalysts possess the potential to achieve both catalytic efficiency and materials recyclability. The research into superparamagnetic electrocatalysts may open new avenues for sustainable energy conversion technologies.
4. To navigate the design of high-entropy catalysts with optimized spin configuration. With the emergence of machine learning and artificial intelligence techniques, there is a growing interest in the exploration and design of high-entropy electrocatalysts. Excitingly, the incorporation of a diverse array of elements into a crystal structure can lead to significant changes in spin configuration. Therefore, understanding how different elements influence the spin configuration of high-entropy catalysts is crucial for establishing theoretical frameworks that support discoveries driven by data science. To achieve this, creating a comprehensive database is essential. This necessitates the adoption of autonomous laboratories to expedite the synthesis and analysis of materials, which, in turn, could lay the groundwork for accelerated innovation in high-entropy electrocatalyst design.
5. To develop more specific theoretical methods to reflect the experimentally observed spin phenomena. Spin generally influences the catalytic performance via spin-selected orbital occupation, spin-selected electron transfer, and spin ordering. Although current theoretical approaches can model all these features, they can only simulate the static spin configuration but fail to capture the dynamic transitions between different spin configurations. Under experimental conditions, the spin configurations of electrocatalysts may dynamically respond to changes in the environment, leading to variations in catalytic performance. Therefore, it is crucial to develop molecular-dynamics–related methods to accurately capture the transition processes of spin configuration during electrocatalysis and assess their impact on catalytic activity. Besides, the significant role of cation spin state has been increasingly recognized in various electrocatalytic systems. However, current spin state manipulation in DFT is performed on the entire simulation box rather than targeting specific cation sites. To precisely reflect the spin state at cation sites, it is essential to develop new methods that can specifically localize the spin state to individual atoms.

In summary, spin-dependent electrocatalysis plays critical roles in numerous energy conversion reactions. The effectiveness of spin-dependent electrocatalysis is subjected to the spin configurations within the electrocatalysts. To comprehensively understand the spin-dependent mechanism in electrocatalysis and enable its integration into sustainable energy systems, it is imperative to develop corresponding characterization technologies to visualize the spin configurations and strategic approaches to fine-tune them. Furthermore, spin-dependent catalysis is currently focused primarily on fundamental research, with its practical applications remaining largely unexplored. To advance towards industrial application, it is crucial to assess the extent to which this effect can enhance *operando* catalytic efficiency. For instance, quantifying the reduction in energy consumption compared to water electrolysis without a magnetic field would be necessary. Therefore, advancements in these specific areas are essential to harness spin-dependent electrocatalysis into industrial applications, which will ultimately contribute to the establishment of a sustainable energy infrastructure.
